# A Glimpse into the AI-Driven Advances in Neurobiology and Neurologic Diseases

**DOI:** 10.3390/biomedicines12061221

**Published:** 2024-05-31

**Authors:** Wu Qiu, Hulin Kuang

**Affiliations:** 1 School of Life Science and Technology, Huazhong University of Science and Technology, Wuhan 430074, China; wuqiu@hust.edu.cn; 2 Hunan Provincial Key Lab on Bioinformatics, School of Computer Science and Engineering, Central South University, Changsha 410083, China

Recent developments in AI, especially in machine learning and deep learning, have opened new avenues for research and clinical practice in neurology. These technologies have demonstrated remarkable proficiency in analyzing complex datasets, identifying patterns that elude human observers, and offering insights into the intricate mechanisms of neurologic diseases. From enhancing diagnostic accuracy to personalizing treatment protocols, AI’s impact is both profound and far-reaching.

This Special Issue has sought to highlight the significant strides made in the application of AI across various aspects of neurobiology and neurological diseases. It has brought novel methodologies to light, explored the therapeutic potential of AI-driven interventions, and showcased innovative research that leverages AI to tackle longstanding challenges in the field.

The ten papers included in this Special Issue collectively contribute to the rapidly evolving field of AI in neurobiology and neurological diseases, highlighting diverse applications of AI technologies ranging from diagnosis to treatment and prevention strategies. Key themes and findings from the papers include the following:

Advanced Diagnostic Techniques: Several studies present AI-driven methods for enhancing diagnostic accuracy in neurology. For example, novel CNN models are used for the identification of ischemic regions in stroke patients [1] and the prediction of the histologic grades of meningiomas from MRI scans [2]. These approaches significantly improve upon traditional methods by leveraging the subtle patterns in medical imaging data that are often undetectable to human observers.

Enhanced Predictive Models: Some papers introduce machine learning techniques to forecast disease progression, such as the development of dementia and epilepsy [3]. By analyzing large datasets and utilizing sophisticated algorithms like SVMs and deep learning, these studies offer predictive models that potentially enable earlier and more accurate interventions.

Therapeutic Applications and Treatment Planning: AI is shown to support therapeutic planning and intervention strategies. One study, for instance, explores how AI can guide the administration of treatments in real time by accurately scoring collateral circulation in stroke patients using hybrid CNN and transformer networks [4].

Automated Systems for Clinical Efficiency: Several papers discuss the role of AI in automating clinical processes such as activity recognition in epileptic patients and the detection of neurological abnormalities. These automated systems are designed to enhance clinical efficiency and patient monitoring [5,6].

Neurological Mechanisms and Disease Biomarkers: AI’s role extends beyond clinical applications into fundamental research, where it helps elucidate underlying disease mechanisms and identify novel biomarkers. This is evident in studies that use AI to analyze EEG patterns and neuroimaging data to gain deeper insights into brain function and disorders [7,8].

This Special Issue marks a significant step forward in our journey toward harnessing the full potential of AI in neurobiology and neurological diseases. There is a need to further refine AI models to increase their accuracy, reliability, and applicability across different populations and disease states. This includes improving the interpretability of AI models to better understand the biological underpinnings of their predictions, integrating multi-modal data sources (e.g., imaging, genetic, and clinical data) for comprehensive disease profiling, and exploring the potential of AI in predicting disease onset and progression, as well as response to treatment.

The exploration of AI’s capabilities in neurology is just beginning. Future research should focus on refining AI algorithms for broader clinical application, developing interoperable systems that can seamlessly integrate with existing healthcare infrastructure, and exploring the ethical implications of AI in patient care. With these efforts, AI holds the promise of significantly transforming neurobiology and the management of neurological diseases. As we continue to explore this frontier, our collective efforts should be guided by a commitment to improving patient care, advancing scientific understanding, and addressing the ethical considerations that accompany the integration of AI into healthcare. The path ahead is both challenging and promising, beckoning us to continue our exploration with diligence, creativity, and an unwavering focus on the future.
